# Management and biosecurity practices on pig farms in the Western Highlands of Cameroon (Central Africa)

**DOI:** 10.1002/vms3.211

**Published:** 2019-11-04

**Authors:** Marc K. Kouam, Manjeli Jacouba, Junior O. Moussala

**Affiliations:** ^1^ Department of Animal Production Faculty of Agronomy and Agricultural Sciences Dschang Cameroon; ^2^ Center for Research on Filariases and other Tropical Diseases (CRFilMT) Yaoundé Cameroon

**Keywords:** African swine fever, biosecurity, erysipelas, Menoua, pig husbandry

## Abstract

African swine fever (ASF), erysipelas and many other infectious and parasitic diseases have seriously compromised the future of pig industry in the Western Highlands of Cameroon. Since implementation of biosecurity measures (BM) is known to reduce the risk of disease transmission, the objective of this study was to describe the pig farming management system as well as the biosecurity practices on pig farms in the Western Highlands of Cameroon. Therefore, 97 farms were investigated using a face‐to‐face interview‐based questionnaire. Biosecurity practices were divided in three components: isolation, traffic control and sanitation. The results revealed that the majority of farms were extensive (73.22%), farrow‐to‐finish farms (59.79%) and essentially raising crossed‐bred (72.75%). The most practiced BM regarding ‘isolation’ were as follows: maintenance of the minimum distance between farms (56.06%) and dispatching of animals of same age in the same room (97.16%); for ‘traffic control’, the measures included the following: assignment of specific tools and equipment (96.86%) to a specific piggery; concerning ‘sanitation’, daily cleaning (97.06%), as well as using disinfectants (89.13%) were mostly implemented. The measures less implemented for ‘isolation’ included fencing (11.83%), compliance with the all‐in all‐out principle (10.11%), use of specific clothing (6.03%) and quarantine (7.69%); for ‘traffic control’, the less adopted measures comprised visitor hands washed before animal handling (11.65%), respect of linear flow principle (13.52%). Concerning ‘sanitation’, these measures included functional footbath (29.90%), processing of drinking water (27.84%) and cleanout (18.14%). The biosecurity level was low, intermediate and high for 73.71, 21.55 and 4.73% of farms, respectively. This low level suggests that ASF and other diseases are likely to remain endemic. The most important measures of concern and to improve are as follows: not feeding kitchen waste to pigs; keeping other livestock species away from pigs; fencing pig barn; keeping newly arrived animals in quarantine, not exchanging boars; not selling sick animals.

## Graphical abstract

Management practices on pig farms in the Western highlands of Cameroon are diversified. Biosecurity measures fall within “isolation”, “traffic control and “saniation”, the three components of biosecurity. The biosecurity level was low, intermediate and high for 73.71, 21.55 and 4.73% of farms respectively suggesting that overall, the majority of farms had a poor biosecurity level., The endemic nature of African swine fever and erysipelas is probably due to low biosecurity.

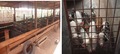

## 1 INTRODUCTION

Pig production has been increasing in the world, with animal number growing from 856 241 to 977 021 thousand heads in 2000 and 2013, respectively (FAO, 2015). The production in Cameroon is following the world trend (from 2 440 404 heads in 2010 to 2 896 271 heads in 2012) due to high demand resulting from the growing population. Though the country is the largest pig producer in Central Africa (MINEPIA, 2009), the demand is not satisfied due to a number of constraints including poor technical inputs in the production system (untrained personnel, old, defective, and worn equipment, inappropriate building among other), poor feeding (low standard feed and lack of feed supplement) and poor health (MINEPIA, 2009). Among the disease challenges, African swine fever (ASF) and erysipelas alone are responsible for heavy losses due to outbreaks that occur almost every year in Cameroon (MINEPIA, 2009). Other infectious diseases reported to occur include hog cholera, porcine encephalomyelitis, Aujeszky's disease, enteritis, transmissible gastroenteritis, porcine encephalomyelitis, erysipelas, dysentery, pasteurellosis, tuberculosis and salmonellosis (MINEPIA, 2009). Parasitic diseases (Strongylid parasites, coccidia, *Strongyloides ransomi Acaris suum*, *Metastrongylus* sp., *Trichuris suis, Macracanthorhynchus hirudinaceus* and so on) (Kouam, Ngueguim & Kantzoura, 2018) are not the least, causing considerable economic losses due to reduced weight gain, litter size, poor growth rates, visceral organ condemnation at slaughter and deaths (Kauffman, 1996;Pitman, 2010;Stewart & Hale, 1988). Thus, one of the important measures to increase pig productivity should be in the area of disease control. Due to the large number of diseases in pigs, implementation of biosecurity measures (BM) in pig production is of paramount importance. For instance, the prevalence of porcine reproductive and respiratory syndrome and *Mycoplasma hyopneumoniae* has been associated with the level of biosecurity at farm level (Austin, Weigek, Hungerford, & Biehl, 1993;Lambert, Arsenault, Polkak, & D’Allaire, 2012). Reversely, several pig diseases (intestinal and miscellaneous diseases) have been successfully controlled through the respect of biosecurity practices in some countries (Wallgren, 2009).

Nowadays, the implementation of BM is regarded as a powerful tool in the control of diseases on the farm; its main advantage is the potential to keep pathogens off the farm and to prevent pathogens from spreading to other farms. Biosecurity can be defined as a set of management practices or measures to prevent introduction and spread of pathogens within and between farms (Fasina, Lazarus, Spencer, Makinde, & Bastos, 2012;Gueye, 2008;Gunn, Heffernan, Hall, McLeod, & Hovi, 2008). In pig production specifically, biosecurity is defined as ‘the implementation of measures that reduce the risk of introduction and spread of disease agents; it requires the adoption of a set of attitudes and behaviours by people to reduce risk in all activities involving domestic, captive/exotic and wild animals and their products’ (FAO/ OIE/ World bank, 2010). BM are divided into three components: isolation, traffic control and sanitation (Cardona & Kuney, 2001;FAO/ OIE/ World bank, 2010). Isolation can be regarded as measures related to physical barriers (fence, showers or footbath) and distance between farms in order to limit contacts between infected animals and contaminated objects with disease‐free farms (FAO, 2008). Traffic control can be considered as the restriction of feedstuff, human, equipment and animal movement onto the farm (FAO, 2008). Sanitation refers to the cleaning and disinfection of animal housing, people material and equipment (Cardona & Kuney, 2001).

Despite the usefulness and impact of BM adoption, no information to assist policymakers in pig industry is available regarding the level of biosecurity implementation in pig farming in the Western Highlands of Cameroon where a number of infectious and parasitic diseases occur (MINEPIA, 2009). Therefore, the overall aim of this study was to describe the pig production management system as well as the associated biosecurity practices in the Western Highlands of Cameroon. Specifically, the three main objectives of this study were (a) to provide the general characteristics of pig farming, (b) to characterize the biosecurity practices and (c) to assess the biosecurity level of pig farms.

## 2 MATERIALS AND METHODS

### 2.1 Study area

**Figure 1:**
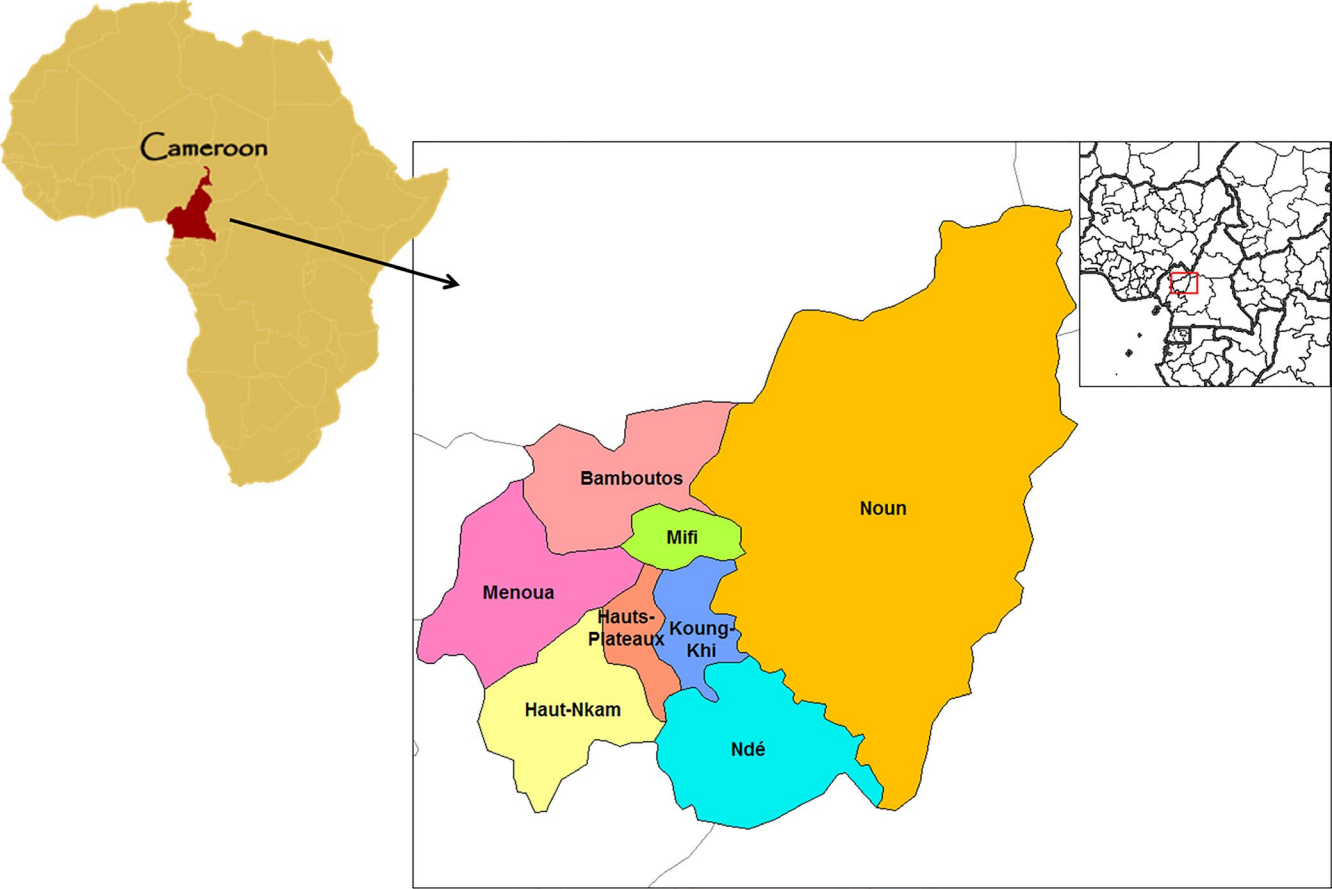
Map of the West Region of Cameroon showing Menoua Division

The study was carried out from May to July 2017 on pig farms located in Menoua Division of the Western Highlands of Cameroon. The subdivisions, Dschang, Fokoue and Penka Michel within the Menoua Division, were chosen due to the importance of pig farming in these locations, as advised by the local veterinary health officials. The area lies between longitude 9°49'–10°20’ East of the Greenwish meridian and latitude 5°17’–6°22’ North of the equator (Figure 1). The region is characterized by a typical climate with two main seasons, the dry season ranging from November to mid‐March and the rainy season which prevail from mid‐March to October. Temperature ranges between 15° and 24°C (IRAD, 2012). Livestock species include pigs, small ruminants, cattle, cavies and poultry. The Western Highlands are one of the largest pig production areas of the country and one of the foci of ASF outbreak in the country (MINEPIA, 2009;MINEPIA/FAO, 2009).

### 2.2 Questionnaire design

The questionnaire consisted of closed question sets (the tables in this manuscript include all the questionnaire items) related to production characteristics (farm size, breed and production system, among others) and biosecurity components (isolation, traffic control and sanitation items). Questions were constructed based on the epidemiology of common pig diseases in Cameroon, with special focus on ASF and erysipelas (MINEPIA, 2009). Before starting the present study, the questionnaire was tested. Testing of the questionnaire was carried out by interviewing a sub‐sample of farmers (*n* = 15) in the study area to check the accuracy and clarity of questions, and whether some questions were missing or redundant. Adjustments were made accordingly.

### 2.3 Selection of farms

In absence of the official registry of pig farms, farms were first located with the help of the local veterinary health officials. The next farms were located using the snow ball technique where the previously located farmer helped to identify the next farmer and so on. The process continued until no new farm could be found within the study area. The survey with the aid of a questionnaire was carried out through a face‐to‐face interview between the researcher and the farmers and through the personal observations of the researcher.

### 2.4 Determination of the adoption level of biosecurity measures by farmers

The adoption level of a given measure was obtained by dividing the total number of farms applying that measure by the total number of farms; the ratio was expressed as a percentage.

### 2.5 Determination of the overall observance of biosecurity measures on the farm

The overall observance is the ratio of applied BM to the required measures expressed in percentage (Racicot & Vaillanourt, 2009). If the ratio is equal or less than 25%, the biosecurity level on the farm is qualified as low. If the ratio falls between 26% and 74%, then the adoption level of BM is intermediate, and the biosecurity level is referred to as intermediate. If it is equal or more than 75%, then the biosecurity level is good (Racicot & Vaillanourt, 2009).

### 2.6 Statistical analysis

The responses obtained were subjected to descriptive statistics (frequency and mean). Data were analysed using the SPSS statistical package (version 13.0, SPSS Inc., USA).

## 3 RESULTS

### 3.1 General characteristics of pig husbandry

A total of 97 farms were identified and visited in Menoua Division (41 [42%] in Dschang, 32 [33%] in Fokoue and 24 [25%] in Penka Michel subdivisions). The general pig husbandry characteristics in the study area are shown in Table 1. Most farms were extensive (73.22%) and 4.33% was intensive. Most farms were farrow‐to‐finish farms (59.79%) while farrower and grower‐finisher farms almost shared the same proportion (22.67 and 17.53%, respectively). The most frequently used breed was crossed breed (72.75%). The work force was divided into three categories (family, paid workers, and both family and paid workers) but family was by far the greatest man power (80.10%). Most farmers (88.65%) used concomitantly concentrate and kitchen waste as feedstuff. Only 11.34% of farmers only gave concentrate to pigs while none exclusively gave kitchen waste to animals. The mean age at first farrowing was 8.70 ± 1.33 months and piglets were weaned within 52.39 ± 7.80 days. The average flock size was 16.87 ± 11.04 pigs and the number of litter per year was 2.

**Table 1:** General characteristics of farms in Menoua Division

Characteristics	Subdivisions			Total (*N* = 97)
	Dschang (*n* = 41)	Fokoué (*n* = 32)	Penka Michel (*n* = 24)	
	%	%	%	%
Husbandry system^a^				
Extensive	69.25	72.64	77.78	73.22
Semi‐intensive	24.89	22.55	19.89	22.45
Intensive	5.86	4.80	2.33	4.33
Production type				
Farrower	19.51	21.88	29.17	22.68
Grower‐finisher	17.07	15.63	20.83	17.53
Farrow‐to‐finish	63.41	62.50	50.00	59.79
Breed				
Improved local breed	15.54	10.05	18.16	15.25
Crossed breed	73.45	74.26	70.55	72.75
Exotic breed	11.01	13.69	9.95	11.55
Man power				
Family	83.17	81.88	72.50	80.10
Employees	11.95	15.00	23.33	15.77
Family and employees	4.88	3.13	4.17	4.12
Feedstuffs				
Kitchen waste only	0.00	0.00	0.00	0.00
Concentrate	14.63	3.13	16.67	11.34
Both	85.37	96.88	83.33	88.65
Age at first farrowing (month)	9.45 ± 1.52*	8.76 ± 0,85	9.2 ± 1,20	8.7 ± 1.33
Weaning age (days)	52.25 ± 7.66	53.28 ± 7.79	51.95 ± 7.93	52.39 ± 7.80
Herd size	17.14 ± 11.62	21 ± 9.89	15.21 ± 10.67	16.09 ± 11.04
Annual farrowing number	2 ± 00	2 ± 00	2 ± 00	2 ± 00

Note

*N* = total number of farms. *n* = number of farm per subdivision. *Values in the table are in percentage, except for continuous variables (age at first farrowing, weaning age, herd size and annual farrowing number) which are presented as mean ± standard deviation.

a extensive system = animals of relatively small number (local and/or crossed breed) are permanently penned and feed on agriculture by‐products and kitchen wastes; semi‐intensive system = crossed bred animals are permanently penned in piggeries with a roughcast floor, feed on kitchen waste, agricultural by‐products and often industrial feed; intensive system = animals are improved breeds, indoors, in high number; the piggery is a modern building; feedstuff is exclusively industrial; management system is modern

### 3.2 Implementation of biosecurity measures

#### 3.2.1 Biosecurity measures related to the ‘isolation’ component

More than half of the farms (55.08%) were located in less than 500 m from the main road, whereas 51.06% complied with the minimum distance of 500 recommended between two farms. Only 11.83% of farms were fenced and none had a sign board forbidding access to piggeries for visitors and pet animals. Having other animal species on the farm was almost a standard as 91.92% of farms also kept domestic animals (small ruminants, fowl, dog, cats and cavies among others). Just 10.11% of farms adhered to the all‐in all‐out principle (all the animals that enter the farms are also removed at the same time to avoid contact between two different batches of animals). Use of specific coveralls and boots as well as quarantine of new animals was applied by 6.03 and 7.69% of farmers. The vast majority (97.16%) of farmers dispatched up pigs in lodges based on their age (the following age group: piglets [2–6 months], growers [6–9 months], and adults > 9 months were separated from each other) and 13.67% of employees also reared pigs at home. Measures concerning the ‘isolation’ component are detailed in Table 2a.

**Table 2:** Biosecurity practices in relation to (a) ‘isolation’, (b) ‘traffic control’, (c) ‘sanitation’ in Menoua Division

Characteristics	Subdivisions			Total (*N* = 97)
	Dschang (*n* = 41)	Fokoué (*n* = 32)	Penka Michel (*n* = 24)	
	%	%	%	%
(a)				
Farms located near the main road (≤500 m)				
Yes	50.55	53.85	60.85	55.08
No	49.45	46.15	39.15	44.92
Distance between two farms (≤500 m)				
Yes	53.90	53.75	39.17	48.94
No	46.10	46.25	60.83	51.06
Farms are fenced				
Yes	16.34	7.50	11.67	11.83
No	83.66	92.50	88.33	88.16
Entry restriction sign board present				
Yes	0.00	0.00	0.00	0.00
No	100.00	100.00	100.00	100.00
Other species are present on the farm				
Yes	90.98	88.13	96.67	91.92
No	9.02	11.87	3.33	8.08
All‐in all‐out system				
Yes	10.39	8.75	11.17	10.11
No	89.61	91.25	88.83	89.89
Use of clean coveralls and boots on farm				
Yes	9.76	0.00	8.33	6.03
No	90.24	100.00	91.67	93.97
New animals are quarantined before entry into the herds				
Yes	5.54	10.88	6.67	7.69
No	94.46	89.12	93.33	92.30
Animals in the flock are provided by many suppliers				
Yes	58.10	65.70	54.27	59.36
No	43.90	34.12	45.82	40.64
Animals of different age in the same box/room				
Yes	2.44	2.65	3.45	2.84
No	97.56	97.35	96.55	97.16
Employees also rear pigs at home				
Yes	9.76	18.75	12.50	13.67
No	90.24	81.25	87.50	86.33
(b)				
Piggeries are built based on linear flow principle				
Yes	17.15	15.10	8.33	13.52
No	82.85	84.90	91.67	86.48
Each employee is assigned to a single building				
Yes	73.17	78.13	79.17	76.29
No	26.83	21.88	20.83	23.71
Visitors are allowed to have contact with pigs without washing their hands				
Yes	14.39	8.75	10.83	11.65
No	85.61	91.25	89.17	88.35
Unsold animals returning from market reintroduced in the flock				
Yes	19.51	34.38	37.50	28.87
No	80.49	65.63	62.50	71.13
Production materials are specific for each piggery				
Yes	100.00	90.00	100.00	96.86
No	0.00	0.00	0.00	3.33
Production materials are exchanged among farms				
Yes	2.44	3.13	8.33	4.63
No	97.56	96.88	91.67	95.37
Farmers use boars from other farms				
Yes	36.59	29.41	25.00	31.96
No	63.41	70.59	75.00	68.04
(c)				
Footbath is functional				
Yes	29.27	31.25	29.17	29.90
No	70.73	68.75	70.83	70.10
Disinfectants are used				
Yes	87.11	90.33	82.99	89.13
No	12.89	9.67	17.01	10.87
Piggeries clean every day				
Yes	97.36	97.19	96.50	97.06
No	2.64	2.84	3.50	2.94
Sanitary lock is present				
Yes	0.00	0.00	0.00	0.00
No	100.00	100.00	100.00	100.00
Draining system is present				
Yes	65.85	75.00	70.00	71.13
No	34.15	25.00	30.00	28.87
Feedstuffs sheltered against rodents				
Yes	48.78	46.88	41.67	46.39
No	51.22	53.13	58.33	53.61
Drinking water is treated				
Yes	26.83	28.13	29.17	27.84
No	73.17	71.88	70.83	72.16
Cleanout				
Yes	19.02	14.38	21.67	18.14
No	80.98	85.63	78.33	81.86
Cadaver management				
buried	79.30	85.7	78.66	81.22
burned	10.54	8.32	4.99	7.95
Given to dogs	2.11	3.12	5.50	3.57
Thrown into dustbin	8.05	2.86	10.85	7.25
Sick animal are sold				
Yes	9.76	15.63	4.17	10.31
No	90.24	84.38	95.83	89.69

*N* = total number of farms. *n* = number of farm per subdivision.

#### 3.2.2 Biosecurity measures related to the ‘traffic control’ component

Practices concerning traffic control are shown in Table 2b. A few farms (13.52%) had piggeries built according to the ‘linear flow’ principle while 76.29% of workers were assigned to a specific building. Building according to the linear flow principle means that the piggery is built in such a way that a farmer or a visitor must move in one direction only, generally from the clean to the dirty area and from the young to the old, without turning back. It was unusual for visitors (11.65%) to wash their hands before touching pigs, whereas 28.87% of farmers reintroduced pigs returning from markets into the herd. Dedication of tools and equipment to a specific piggery within a farm was close to 100% (96.86%). Among farms, exchange of tools and equipment was very uncommon (4.63%). About 32% of farmers borrowed boars from neighbouring farms for breeding purposes.

#### 3.2.3 Biosecurity measures related to the ‘sanitation’ component

Results on sanitation practices (Table 2c) showed that functional footbaths were available in 29.90% of farms, while cleaning on a daily basis and use of disinfectants were practiced by 97.06 and 89.13% of farmers, respectively. Sanitation lock and shoe cleaning post were absent on all the farms. Wastewater draining system, processing of drinking water and cleanout were adopted on 71.33, 27.84 and 18.14% of the farms, respectively. Almost half (46.39%) of the farms had feedstuff protected from rodents.

#### 3.2.4 Overall observance of biosecurity measures

As a whole, the vast majority (73.71%) of farms had a low biosecurity level (adoption rate of BM equal or less than 25%). A very few (4.73%) farms had a good level of biosecurity (Table 3).

**Table 3:** Overall level of biosecurity measures in Menoua Division

Adoption rate (%)	Subdivisions			Total (*N* = 97)
	Dschang (*n* = 41)	Fokoué (*n* = 32)	Penka Michel (*n* = 24)	
	%	%	%	%
[0–25]	71.98	72.65	76.52	73.71
[25–75]	21.64	22.91	20.12	21.55
[75–100]	6.39	4.45	3.36	4.73

Note

*N* = total number of farms. *n* = number of farm per subdivision.

In general, the biosecurity level was poor, irrespective of the biosecurity component. In fact, up to 75.85, 65.85 and 77.46% of farms had a low biosecurity level for the ‘isolation’, ‘traffic control’ and ‘sanitation’ component, respectively, with an adoption rate of BM equal or less than 25%. The highest number of farms (9.74%) with a good biosecurity level (adoption rate of BM equal or more than 75%) was observed within the ‘traffic control’ component. The adoption rate of BM for each component is presented in detail in Table 4.

**Table 4:** Overall level of biosecurity measures according to biosecurity components in Menoua Division

Adoption rate (%)	Subdivisions			Total (*N* = 97)
	Dschang (*n* = 41)	Fokoue (*n* = 32)	Penka Michel (*n* = 24)	
	%	%	%	%
Isolation				
≤25	74.75	75.48	76.83	75.85
[26–74]	20.92	1.85	17.71	19.45
≥75	4.33	4.67	5.46	4.70
Traffic control				
≤25	62.85	65.56	69.25	65.85
[26–74]	27.45	24.15	22.52	24.41
≥75	9.70	10.29	8.23	9.74
Sanitation				
≤25	76.62	78.59	79.48	77.46
[26–74]	20.15	19.82	18.65	20.56
≥75	3.23	1.59	1.87	2.97

Note

*N* = total number of farms. *n* = number of farm per subdivision.

## 4 DISCUSSION

This study described the general characteristics of pig husbandry in an area within the Western Highlands of Cameroon, one of the largest pig production regions of the country (MINEPIA, 2009). The extensive production system was found to be the most predominant. Our result is similar to previous findings by the Ministry of livestock, fisheries and animal industry (MINEPIA, 2011). This may be partly explained by the family attachment to traditions and secondly by the lack of financial means and adequate training. Most farmers were engaged in both piglets production and fattening (farrow‐to‐finish) and the least preferred production option was fattening. This could be due to the fact that piglets are readily sold. The crossed breeds were reared by most farmers (72.75%). This percentage (72.75%) is higher than 56.80% reported by Ndébi, Kamajou, and Ongla (2009) and could be related to the fact that with time farmers have noticed that the mixed breeds are zoo‐technically more efficient than the pure local breeds (heterosis effect). Man power in the study area was essentially made up of family members, confirming the results obtained by other authors (Ndébi et al., 2009) in the Western Highlands of Cameroon. This may be explained by the low income which precludes farmers from hiring off‐family workers. The insufficient income corroborates with the low herd size (16.08 ± 11.04), the production system and the feedstuff (kitchen waste and concentrate) used in most farms (88.65%). Combining kitchen waste with concentrate is more economical than using concentrate alone. However, since kitchen waste is not heated before supply to animals, the risk of disease transmission (ASF, erysipelas and salmonellosis among others) is increased. Indeed, kitchen waste contains many food remains whose origin and safety are unknown even by farmers themselves because these waste are generally collected from public dustbins. These wastes are likely to carry ASF virus from the remains of a contaminated pig because the virus can survive for months in protein‐rich materials (FAO, 2011). The wastes are also likely to carry other viruses (rotavirus, coronavirus and swine influenza viruses), bacteria (*Erysipelothrix rhusiopathiae*, *Salmonella*, *Escherichia coli*) and parasites (*Cryptosporidium* spp., coccidia, helminths). Kitchen wastes are serious hazards in pig farming in the country in general because feeding pigs with kitchen waste is a common practice countrywide. Farmers need to be sensitized on the danger of such a practice. The mean farrowing age observed was 8.70 ± 1.33 months, which was lower than 11.67 ± 35 months reported in Douala (Kouamo, Tankou, Zoli, Bahn, & Ngo Ongla, 2015) or 10.90 ± 2 months reported in Garoua (Mopate‐Logtene et al., 2009) in Northern Cameroon. The discrepancy is probably due to the difference in the genetic material since the dominant breed in Garoua is the local breed characterized by a lower performance, in contrast to the improved mixed breeds found in the Western Highlands of the country. The mean weaning age (52.39 ± 7.80) was higher than 35 days obtained in Kounden (Center region of Cameroon) (Gweth, 2016) but the discordance should be related to the difference in husbandry conditions, as the rearing of pigs in Kounden was performed in a monitored station under better biosecurity conditions.

Concerning BM and specifically the ‘isolation’ component, half of the farms visited were located around a main road while the minimum distance required between two farms was not respected by 48.94% of farms; this short distance is likely to increase the risk of disease transmission on farms. This obviously is against the FAO/OIE/Work Bank (2010) recommendations, which require a minimum distance of 500 m between farms and from a main road (Pritchard, Dennis, & Waddilove, 2005;Vangroenweghe et al., 2009). Farmers are probably unaware of these measures. In addition, there are no established regulations to guide farmers on the basic BM. A fence was present in only 11.83% of farms and all the farms visited did not have a sign board or any physical notice preventing access on farms for visitors and companion animals. These results disagree with the FAO/OIE/Work Bank (2010) recommendations and with other findings in Nigeria where 84.60% of farms were found to be fenced (Maduka, Igbokwe, & Atsanda, 2016). These results may be due to the fact that the majority of farms belong to the extensive management system. Other animal species were found in 91% of farms, which is a serious biosecurity problem since cross transmission of pathogens between different species has been demonstrated (Pensaert, Ottis, Vandeputte, Kaplan, & Bachmann, 1981;Wall et al., 1995). The poor situation is worsened by the fact that the fence was absent around most piggeries, making a contact between pigs and wildlife possible. Some diseases/pathogens that can be transmitted from other animals to pigs or vice versa include: *Bordetella* spp*.*, avian tuberculosis, *Salmonella* spp*.* and avian influenza (Anonymous, 2010;Pensaert et al., 1981;Vangroenweghe et al., 2009) from birds to pigs; classical swine fever (Fritzemeier et al., 2000) and Aujesky's disease (Artois et al., 2002) from wild boar to pigs; *Salmonella* Typhimurium between pigs, poultry and ruminants (Wall et al., 1995). The all‐in/all‐out principle was followed by 10.11% of farms only. Though it is one of the most important BM in the sense that all animals of the production round are removed and the piggeries are clean and disinfected before the arrival of a new batch, the adoption rate of this measure was low and in contrast with the FAO/OIE/Work Bank (2010) recommendations. In fact, this principle was shown to break the infectious cycle of pathogens from one production round to another (Clark, Freeman, Scheidt, & Knox, 1991). Similarly, there was a poor implementation of quarantine area (7.69%). This might be explained by the fact that the vast majority of farmers have not been trained in pig farming and in biosecurity practices. Also, only 6.03% of farmers used farm‐specific clothes and boots, which is very risky as humans can act as mechanical vectors of disease to pigs. In this case, transmission may occur through leftovers of urine and faeces from infected animals on footwear and clothing as has been proven through experiments for several germs, among which *E. coli* (Amass et al., 2003) and classical swine fever virus (Ribbens, Dewulf, Koenen, Maes, & Kruif, 2007). A vast majority of farmers (97.16%) kept animals of same age‐group in the same unit (piglets, growers and adults separated and kept in different units). This is a good measure that reduces the risk of infections spread. Another good measure is the fact that most employees did not raise pigs at home; this result is probably explained by the fact that most farms (80.10%) had family members as the main work force.

As far as ‘traffic control’ is concerned, implementation of BM appears to be related to the husbandry system. The low adoption level (13.52%) of the linear flow principle is consistent with the low number of farms under intensive and semi‐intensive system (4.33 and 22.45%, respectively). The main advantage of the principle is that the ‘clean area’ cannot be contaminated by a pathogen from the dirty area, unless rodents and insect control is poor or the clean road is used by unclean and non‐disinfected vehicles (Neumann, 2012). The majority of farms assigned a specific employee to a specific piggery. As the number of visitors should be limited on a farm, so also should the number of workers per animal barn be limited. Otherwise, an employee taking care of several buildings can transmit pathogen from one building to another (Kapperud et al., 1993). This result is explained by the fact that most farms are made up of a single animal barn. In a few farms (11.65%), visitors washed their hands before touching an animal; again, this finding is consistent with the low number of farms under semi‐intensive and intensive husbandry system where the biosecurity level has been reported to be higher than in extensive system (FAO/OIE/Work Bank, 2010); the fact that only this percentage of farms adopted this measure is a serious biosecurity flaw in the area. In general, production tools and equipment were specific for a piggery, which is in line with the fact that these tools and equipment were exchanged among farms only by a very limited number of farmers. However, the protective effect of these measures was jeopardized by the habit to exchange boars among 31.96% of farms for reproduction purposes. It is well established that introduction of new genetic materials (semen) or animals from different source herds increases the risk of disease introduction into the pig farm (Hege, Zimmermann, Scheidegger, & Stärk, 2002;Lo Fo Wong, 2004;Pritchard et al., 2005). Exchange of animals among farms is one of the most rapid ways for disease dissemination because an animal may apparently look healthy while it is a chronic, asymptomatic carrier of pathogens. Once introduced into a farm, such an animal becomes the transmission source of diseases to naïve animals. For instance, pigs with the chronic form of ASF can live several months (FAO, 2011); as long as they live, they will continue to be a hazard for pig farms because the virus is likely to be shed via their secretions and excretions. Therefore, the widespread habit of boars exchange observed among farmers is of major concern. The recurrent outbreaks of the so‐called ‘red diseases’ (this expression is used in Cameroon to designate these common diseases of pigs that clinically exhibit skin red colour) in Cameroon might be maintained through this devastating practice. Exchange of boars without prior assurance about the disease‐free status of both the boars and the sows should be strongly avoided. An alternative solution for reproduction could be the vulgarization of the artificial insemination technique, which is already practiced on some limited number of farms in the country.

The ‘sanitation’ component appeared to be closely linked to the production system. Footbaths were available in only 29.90% of farms, which can be explained by the small number of farms under semi‐intensive and intensive system. Footbath is essential for disinfection of footwear and should be renewed on a regular basis. This level is low and in disagreement with the findings (58.00%) obtained by Anne, Isabelle, and Mai (2010). Though footbaths appear of little effectiveness against ASF due to the fact that the boots are not the only wears that may carry virus, they may reduce the risk of transmission if they are correctly used. Use of disinfectant and daily cleaning was implemented in the large majority of farms, suggesting that most farmers understand the need to keep the farms cleaned and disease‐free. Also, most producers had the slurry draining system functional on their farm confirming their good disposition to protect their farms from diseases. The cleanout was adopted only in 18.14% of farms. This poor adoption level should be due to the fact that most farms under extensive system do not follow the all‐in all‐out principle, such that animals are always present on the farm.

As a whole, the biosecurity level on farms in the study area was low, with up to 73.71% of farms having an adoption rate equal or less than 25.00%. The low biosecurity level is probably related to the fact that the majority of farms operated under extensive system. Our finding is in accordance with the FAO/OIE/Work Bank (2010) observations. Considering the three components of biosecurity, the highest percentage of farms with an adoption rate of BM equal or more than 75.00% was not greater than 10.00%, with the ‘traffic control’ component enjoying the first position (9.70%), followed by the ‘isolation’ (4.70%) component and finally the ‘sanitation’ component occupying the last position (2.97%). This low level of biosecurity observed for all the components suggests that farmers do not have any understanding of biosecurity principles in pig farming, and that steps (training and sensitization) should be taken to fill the gap.

In conclusion, the production system in the Menoua Division was dominated by the extensive pig production system. The low implementation level of BM suggests that farmers still have a long way to go and that government officials should handle the issue of biosecurity in pig farming sectors very seriously. With these alarming results, it is not surprising that ASF has become endemic in pig production areas of the country and is still causing a tremendous harm to the pig industry. Parasitic diseases as well as diarrhoea have also become an important health constraint in pig production probably as a result of the very poor biosecurity level on the farms. The most important measures of concern and to improve are; not feeding kitchen waste to pigs; keeping other livestock species away from pigs; fencing pig barns; keeping newly arrived animals in quarantine, not exchanging boars; not selling sick animals. Other countries with similar husbandry systems might probably face the same flaw in biosecurity practices. Future works will focus on the relationship between biosecurity and production performance, incidence of different diseases and culling rates in the area.

## CONFLICT OF INTEREST

The authors declare that they have no conflict of interest.

## ETHICAL STATEMENT

N/A. The study is not reporting results from an experiment on animals or humans.
